# HDAC Inhibitors for the Therapy of Triple Negative Breast Cancer

**DOI:** 10.3390/ph15060667

**Published:** 2022-05-26

**Authors:** Cristina Maccallini, Alessandra Ammazzalorso, Barbara De Filippis, Marialuigia Fantacuzzi, Letizia Giampietro, Rosa Amoroso

**Affiliations:** Department of Pharmacy, University G. d’Annunzio of Chieti-Pescara, Via dei Vestini, 31, 66100 Chieti, Italy; alessandra.ammazzalorso@unich.it (A.A.); barbara.defilippis@unich.it (B.D.F.); marialuigia.fantacuzzi@unich.it (M.F.); letizia.giampietro@unich.it (L.G.); rosa.amoroso@unich.it (R.A.)

**Keywords:** anticancer, benzamide, chimeric compounds, hydroxamate, histone deacetylase, inhibitor, triple negative breast cancer

## Abstract

Triple negative breast cancer (TNBC) is an urgent as well as huge medical challenge, which is associated with poor prognosis and responsiveness to chemotherapies. Since epigenetic changes are highly implicated in TNBC tumorigenesis and development, inhibitors of histone deacetylases (HDACIs) could represent a promising therapeutic strategy. Although clinical trials involving single HDACIs showed disappointing results against TNBC, recent studies emphasize the high potential impact of HDACIs in controlling TNBC. In addition, encouraging results stem from new compounds designed to obtain isoform selectivity and/or polypharmacological HDAC approach. The present review provides a discussion of the HDACIs pharmacophoric models and of the structural modifications, leading to compounds with a potent activity against TNBC progression.

## 1. Introduction

Breast cancer (BC) is the most prevalent cancer in females worldwide. In 2020, 2.3 million women were diagnosed with BC and 685,000 deaths were recorded [1], which represents a public health issue. Among the BC subtypes, the hormone receptor (HR) positive is the most common, followed by the human epidermal growth factor receptor 2 (HER2) positive and the so-called triple negative (TN) phenotype. Triple negative breast cancers are the most aggressive subtype, since they present high-grade invasiveness, high tendency to give rise to distant metastasis, and are poorly responsive to the neoadjuvant anthracycline- and taxane-based chemotherapies. Dramatically, the overall TNBC patients’ survival is about 1 year [2]. Therefore, the identification of new therapeutic options to treat this cancer is an urgent as well as huge medical challenge.

**Figure 1:**
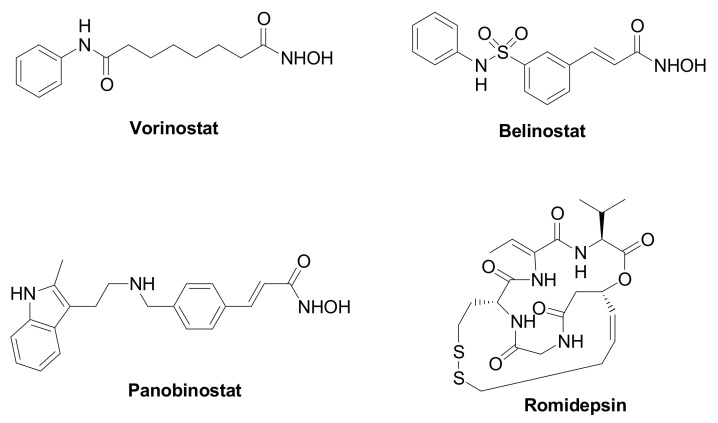
Chemical structure of FDA approved HDAC inhibitors.

Epigenetic changes are highly implicated in TNBC tumorigenesis and development, and they are affected by the chromatin remodeling [3]. Histone deacetylases (HDACs, Table 1) are a family of specialized enzymes responsible for the removal of acetyl groups from the ε-amino group of the proteinogenic histone lysine residues, leading to a compressed chromatin structure and to the consequent gene transcription suppression [4]. Based on the homology with yeast deacetylases, cellular localization, size, and number of active sites, they can be divided into four classes, i.e., Class-I (HDAC isoforms 1, 2, 3, and 8); Class-IIa (HDAC isoforms 4, 5, 7, and 9); Class-IIb (HDAC isoforms 6 and 10); and Class-IV (HDAC isoforms 11). On the other hand, histone acetyltransferases (HATs) are responsible for the inverse modification, i.e., they catalyze the lysine acetylation, leading to chromatin relaxation and active gene transcription [5]. Considering their crucial role in gene transcription and translation, and their overexpression in a variety of human cancers, HDACs have received considerable attention from researchers as therapeutic targets to manage cancer initiation and progression [6]. Different HDAC inhibitors (HDACIs) have been approved by the Food and Drug Administration (FDA), particularly against hematological diseases, such as Vorinostat, Romidepsin, Belinostat, and Panobinostat (Figure 1). Moreover, HDACIs have been investigated for the treatment of solid tumors, including TNBC, although clinical trials involving single agents provided poor results. Nevertheless, recent studies emphasize the high potential impact for HDACIs in controlling TNBC, since these agents can regulate the tumor angiogenesis and metastasis [7,8].

The present review aims to discuss the recent developments in the research of new HDACIs directed against TNBC, with a particular focus on the discovery of chimeric compounds, which simultaneously modulate HDAC and other dysregulated targets as a promising multitargeting approach to manage TNBC.

## 2. HDAC Inhibitors and their Mode of Action against TNBC

### 2.1. Pharmacophoric Model of HDAC Inhibitors

**Figure 2:**
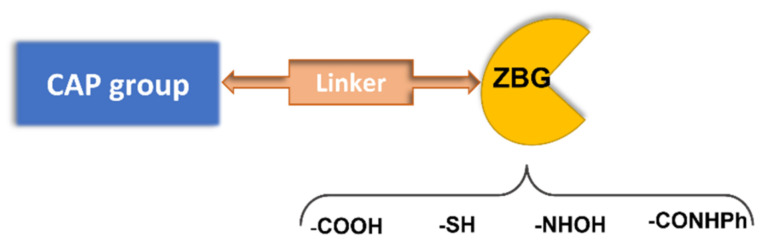
Pharmacophoric model for HDAC inhibitors.

A pharmacophoric model has been established for HDAC inhibitors based on the common structure of the different 11 zinc-dependent isoforms of the HDAC family. The deacetylase domain of these enzymes shows a surface cavity, which is connected to the inner active site by means of an intermediate channel, where the four carbons of the lysine side chain of the natural substrate are accommodated. The active site contains a Zn^2+^ ion, which binds to the histone N-ε acetylated lysine, with the subsequent attack to the N-acetyl group by a water molecule and the production of an N-3 free lysine and acetic acid [9]. In some HDAC isoforms, there is an additional hydrophobic cavity referred to as the “foot pocket”, where acyl side chains longer than the acetyl are accommodated [10]. Based on this enzyme architecture, HDACIs are typically endowed with a zinc binding group (ZBG) and an aromatic tail occupying the entrance area (CAP group), connected by a central lipophilic linker (Figure 2). According to the ZBG, HDAC inhibitors are classified in four classes: carboxylic acids, thiols, benzamides, and hydroxamic acids (Figure 2).

**Figure 3:**
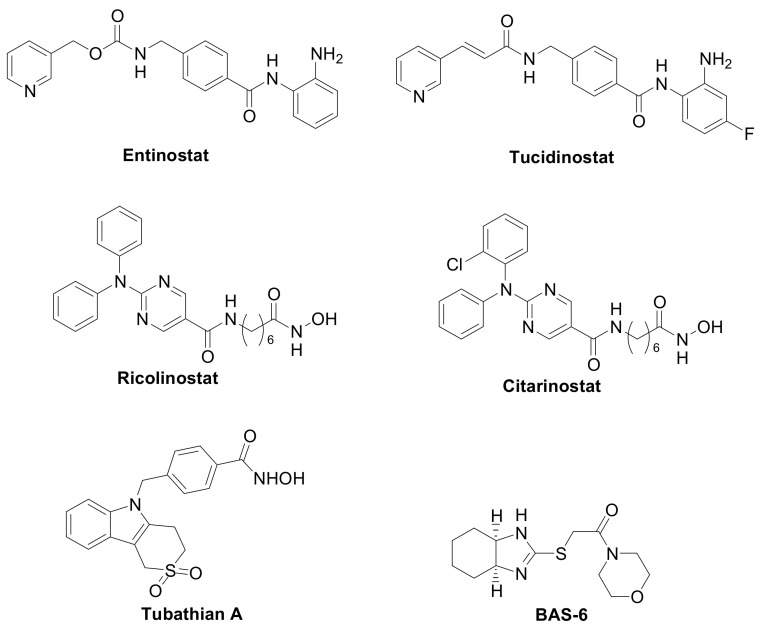
Class-selective HDAC inhibitors.

The first generation of compounds obtained based on this pharmacophoric model, not exploiting the differences among the HDAC isoforms, resulted in pan-HDAC inhibitors that are often associated with numerous side effects. Indeed, the biological consequences of targeting multiple HDACs can be unpredictable, given the pleiotropic activities of these enzymes. Thanks to the increasing availability of crystallographic data and comprehensive SAR studies, significant advances in the research of HDACs isoform-selective inhibitors have been carried out [11,12]. The major differences among the HDACs are found at the entry of the active site and in the channel length and size. Moreover, the presence or the absence of the “foot-pocket” is another important difference among these metallo-enzymes, which could be exploited in the design of selective agents. For example, Entinostat and Tucidinostat are benzamide-based Class-I selective HDAC inhibitors (Figure 3) currently under clinical evaluation in both the US and Europe for the treatment of both cancer and nononcological diseases [13,14]. Ricolinostat and Citarinostat (Figure 3) are hydroxamates, which are quite selective toward the Class-II HDAC6 (HDAC1/HDAC6 selectivity index = 13 and 12, respectively) and are under evaluation against different malignancies [15,16]. In general, new and more selective HDAC6 inhibitors have been disclosed as useful tools to counteract tumor progression [17,18], although it was also reported that the selective inhibition of HDAC6 is not necessarily related to an anticancer activity, as observed for Tubathian A (Figure 3) and related compounds [19]. Therefore, increasing attention is required for the evaluation of HDAC inhibitors as anticancer drugs, especially when used as single agents. In recent years, several researches in the field of HDAC inhibitors have pointed to the hydroxamic acid and benzamide type scaffolds that selectively target HDAC. Currently, hydroxamates hold clinical success with respect to benzamides, with three FDA approved molecules (Vorinostat, Belinostat, and Panobinostat), while only Tucidinostat has been approved by the Chinese FDA to treat patients with recurrent or refractory peripheral T-cell lymphoma [20].

### 2.2. HDACIs Biological Action against TNBC Progression

Both unselective and selective HDACIs have been investigated against TNBC. Additionally, these studies have disclosed the different pathways influenced by HDACIs that trigger the observed antitumoral effects (Table 2).

In general, the pan-HDAC inhibitors Vorinostat and Panobinostat act alone as antiproliferative and pro-apoptotic agents against TNBC cell lines, such as MDA-MB-231, 4T1 and BT-549, by the modulation of multiple pathways and factors. The following factors can be mentioned: the activation of the epithelial-mesenchymal transition (EMT) phenotype and the downregulation of the growth factor FOXA1 [21], the impairment of the homologous recombination repair pathway of DNA [22], the upregulation of tumor suppressor factors p21 and p27, and the downregulation of the survival protein Bcl-2 [23]. Moreover, Vorinostat has the ability to impair BC cell migration and invasion via the inhibition of matrix metallo-proteinase 9 (MMP9) activity [24], both alone and in combination with ionizing radiation.

Interestingly, the Class-I selective HDACI Entinostat, but not pan HDACIs, had the ability to convert ER-negative tumors to ER-positive tumors, sensitizing TNBC to letrozole treatment [25,26]. Moreover, Entinostat demonstrated the ability to upregulate genes involved in the development of the vasculogenic mimicry, i.e., a tumor blood supply system independent from angiogenesis [8]. In addition, a Class-II selective inhibitor directed against the HDAC6, the BAS-6 (Figure 3), selectively kills apoptotic-resistant TNBC cells inducing their glycolytic metabolism alteration [27].

To date, the outcomes obtained in the TNBC management by HDACIs point to the significant potential of these compounds in controlling cancer proliferation and chemo-resistance.

## 3. Recent Developments in the Discovery of HDAC Inhibitors Targeting TNBC

### 3.1. Hydroxamates

In general, the pharmacophore hydroxamate has received significant attention in the field of anticancer drug discovery [32]. Additionally, it is a well-established ZBG in the design of new HADACIs directed against TNBC. Numerous and recently developed hydroxamates are based on the Vorinostat leading scaffold.

**Figure 4:**
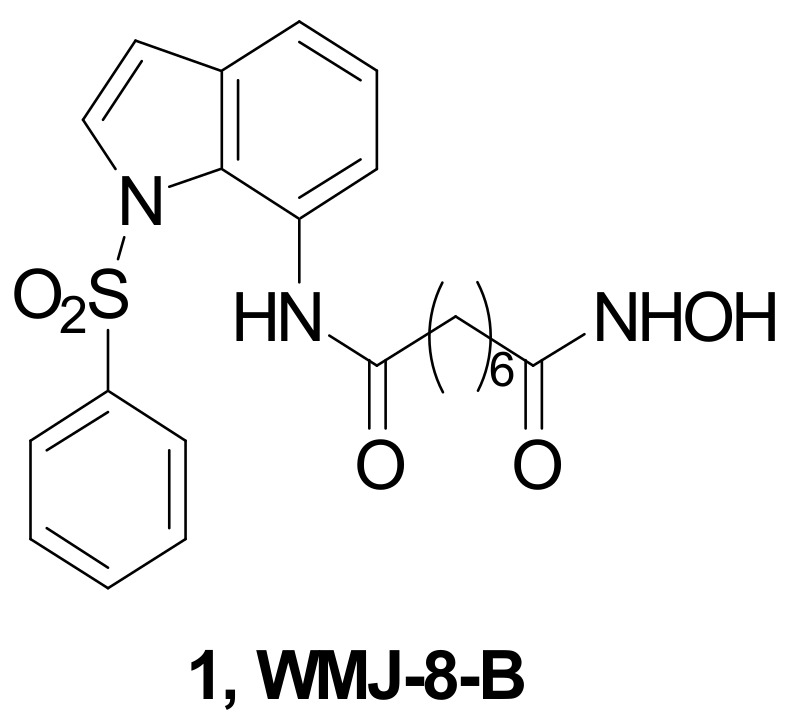
Chemical structure of the HDAC4/6 inhibitor WMJ-8-B.

Compound **1** (WMJ-8-B, Figure 4) is an indolyl-aliphatic hydroxamate, which preferentially inhibits both HDAC4 and HDAC6, exhibiting antiproliferative and pro-apoptotic effects in MDA-MB-231 TNBC cells, through the inactivation of the SHP-1-signal transduction and activators of transcription 3 (STAT3)-survivin pathway and the activation of Sp1-p21 signaling cascade. Moreover, WMJ-8-B had the ability to inhibit the growth of MDA-MB-231 tumor xenografts in vivo.

**Figure 5:**
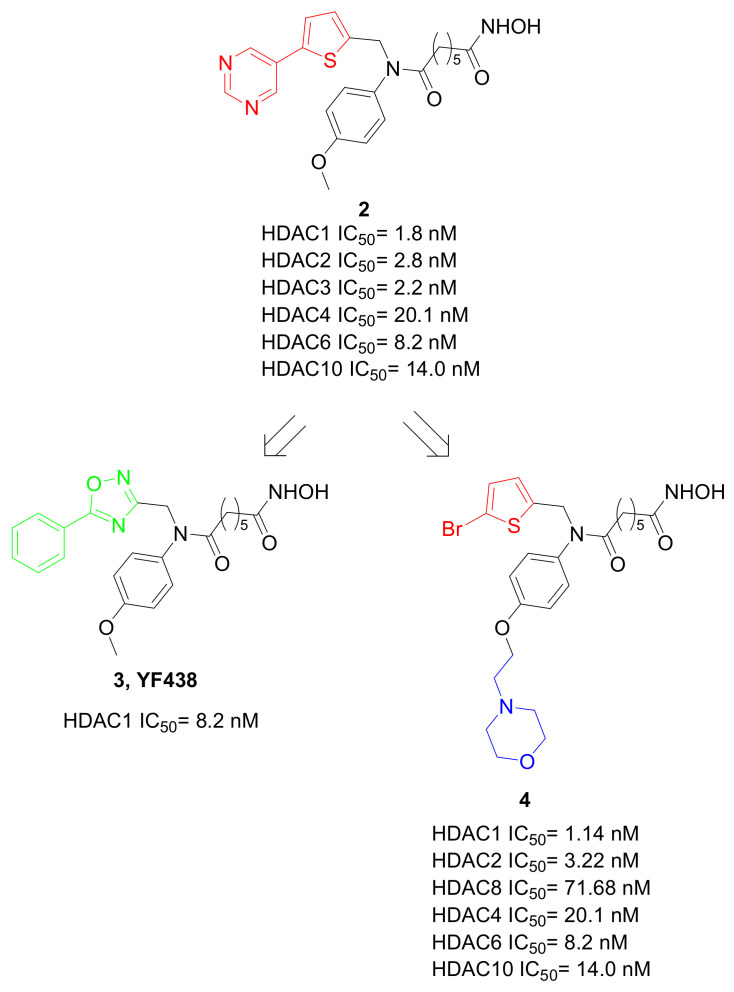
Chemical structures of hydroxamates HDACIs based on a bis-substituted aromatic amide scaffold.

To tailor the isoform selectivity, a library of hydroxamates based on a bis-substituted aromatic amide scaffold was designed [33,34], and the pyrimidinylthiopheneyl derivative **2** (Figure 5) showed a nanomolar IC_50_ toward Class-I-IIb HDACs. This compound counteracted the proliferation and migration of MDA-MB-231 TNBC cells (IC_50_ = 0.48 μM). More importantly, it significantly suppressed tumor growth in a breast tumor xenograft mouse model. The importance of the bis-substituted aromatic amide scaffold for the observed anticancer activity related to the Class-I HDAC inhibition was confirmed by the 1,2,4-oxadiazol-3-yl-aliphatic analogues, from which compound **3** (**YF438**, Figure 5) emerged as the most interesting compound (HDAC1 IC_50_ = 8.2 nM). Very recently, it was reported that this molecule significantly inhibited the cell growth and metastasis of TNBC both in vitro and in vivo, accelerating the degradation of the oncoprotein mouse double minute2 homolog (MDM2) [35]. This study, while confirming the potential anticancer activity of hydroxamic-based HDACIs with branched aromatic tail, suggests that targeting the HDAC1-MDM2 pathway could be a promising strategy for the therapy of TNBC. Further modifications of the compound **2** scaffold led to a set of thiophene-based N-bis-substituted aromatic amide hydroxamic acids, and molecule **4** (Figure 5) emerged as the most promising derivative [36]. Structure-activity relationship studies showed that the length of the alkoxy chain at the para-position of the phenyl ring, and its substitution with a morpholine or a piperazine moiety, were important to improve the activity of these compounds. Compound **4** was a Class-I-II HDAC inhibitor, and had the ability to impair TNBC growth both in vitro and in vivo, confirming the key scaffold of the discussed branched inhibitors.

**Figure 6:**
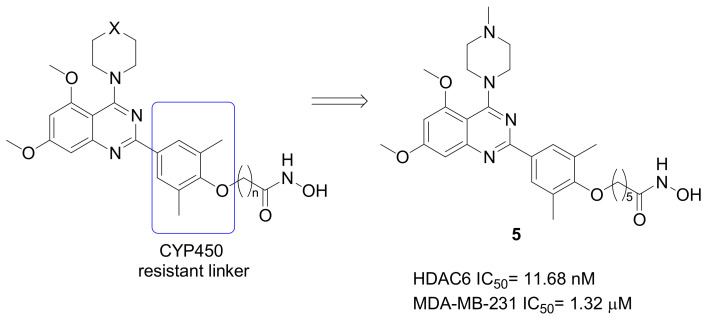
Chemical structures of CYP450 resistant quinazoline-derivatives as Class-IIb HDACIs effective against TNBC.

Class-IIb HDAC inhibitors were identified in a research study aimed to ameliorate the Vorinostat pharmacokinetic profile [37]. Indeed, this molecule has a very short half-life in humans (21–58 min), and the main metabolic biotransformation comprise glucuronidation, alkyl chain *β*-oxidation, and hydrolysis of both hydroxamate and amide moieties [38]. Considering the Vorinostat metabolic unstable amide bond and based on the well-accepted pharmacophoric model of HDACIs, new compounds were designed showing the following main characteristics (Figure 6): (i) An ether bond connected the linker to the CAP group; (ii) a 3,5-dimethylphenyl group was inserted to sterically protect the alkyl linker from the CYP450 metabolism; (iii) a quinazoline core was introduced as the CAP group. Compound **5** (Figure 6) was the most interesting molecule, demonstrating a potent HDAC6 and HDAC10 inhibition (IC_50_ = 11.68 nM and 13.24 nM, respectively), as well as the inhibition of MDA-MB-231 cell proliferation (IC_50_ = 1.32 μM) and migration, both in vitro and in vivo. In particular, this compound induced cell apoptosis and autophagy, and demonstrated improved in vivo antitumor efficacy compared with Vorinostat.

**Figure 7:**
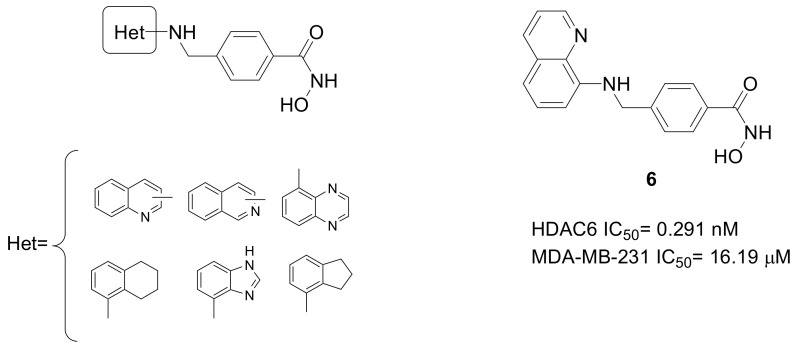
Chemical structures of quinoline-derivatives as selective HDAC6 inhibitors directed against TNBC.

Considerable results in terms of isoform selectivity were obtained from a study investigating (N-Hydroxycarbonylbenylamino)quinolines as HDAC6 inhibitors (Figure 7) [39]. Compound **6** inhibited HDAC6 with good potency (IC_50_ = 0.291 nM) and an excellent degree of selectivity with respect to the other isoforms, comprised between 376-folds (vs. HDAC11) and 42,955-folds (vs. HDAC2). This compound, which was in vivo effective against the progression of multiple myeloma, later demonstrated the ability to suppress TNBC metastasis, impairing cell motility by the inhibition of cofilin-F-actin pathway and the cortacin/F-actin binding, in addition to blocking HDAC6 and increasing heat shock protein 90 (Hsp90) acetylation.

### 3.2. Benzamides

Benzamide derivatives are emerging as selective HDACIs useful in the treatment of TNBC. These compounds are slow, tight binding, and slow disassembling HDAC inhibitors, and appear to be less subjected to glucuronide conjugation with respect to hydroxamates, accounting for less safety issues [40,41,42].

**Figure 8:**
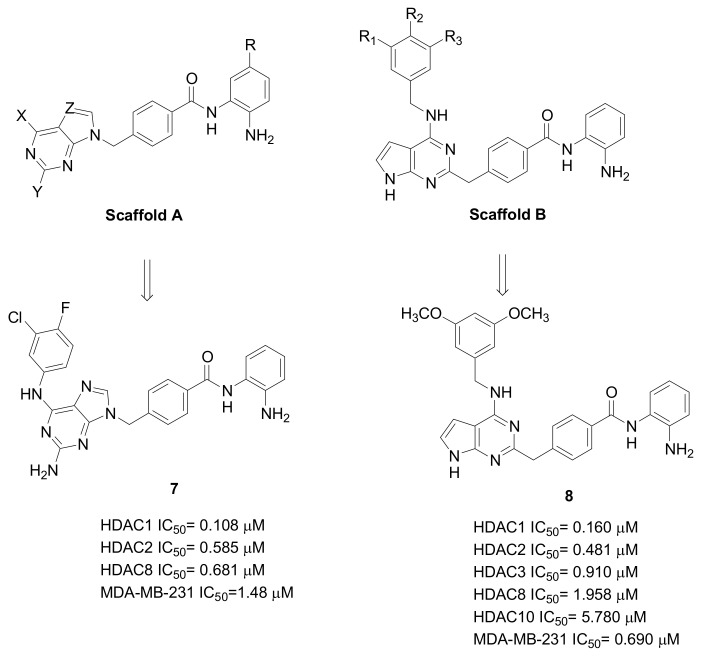
Chemical structures of purine and pyrimidine-based benzamides as Class-I HDACIs with promising activity against TNBC.

Entinostat (Figure 3) is one of the first benzamide-based HDACIs demonstrating promising activity against TNBC development [43]. Indeed, it compromised the tumor-initiating cell (TIC) population as well as mammosphere-forming ability in MDA-MB-231, BT549, and Hs578T cell lines, resulting in reduced tumor formation in vivo. Furthermore, Entinostat treatment impaired the metastatic lung colonization and reduced the expression of miRNA-181a [43]. Moreover, important results have been obtained by Tucidinostat (Figure 3), a Class-I HDAC inhibitor, with specificity in targeting HDAC1, 2, 3, and 10 [44]. MDA-MB-231 and BT-20 cells were treated with Tucidinostat and the results revealed that it suppressed the proliferation, colony formation, and migration of the TNBC cells [45]. Starting from the leading structures of both Entinostat and Tucidinostat, a library of benzamides was recently synthesized, comprising purine or 7H-pyrrolo[2,3-d] pyrimidine core (scaffold A and B, respectively, Figure 8) as the inhibitor CAP group [46,47]. Different Class-I HDACIs were obtained from these scaffolds, which were studied against the proliferation of TNBC cell lines, and compounds **7** and **8** (Figure 8) were the most promising HDACIs directed against TNBC. As for scaffold A, it was observed that the substitution at position 4 of the aniline provided potent antiproliferative effects and the presence of the amino group at position 2 of the purine core is important to determine the observed selectivity of action against HDAC1. Additionally, this innovative HDAC1 inhibitor demonstrated potent in vivo antitumor efficacy in human MDA-MB-231 breast cancer xenograft model. Moreover, compound **8** had the ability to impede microtubule assembly and downregulate tubulin acetylation, triggering the cell-cycle arrest in mitosis.

**Figure 9:**
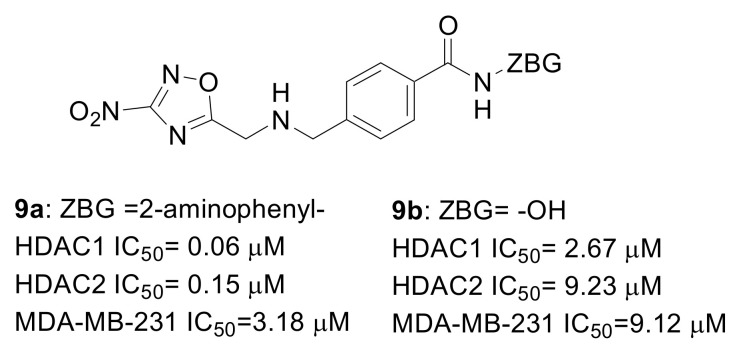
Chemical structure and HDAC inhibition potency of compounds **9a** and **9b** as anti-TNBC agents.

Encouraging results were obtained by a series of 1,2,4 oxadiazole derivatives developed from Entinostat (**9a**, Figure 9), which were HDAC1 and HDAC2 selective inhibitors endowed with antiproliferative low micromolar activity against MDA-MB-231 TNBC cells. Interestingly, the analogue series of hydroxamates **9b** were found to be less potent against HDAC as well as less effective in impairing cancer cells proliferation [48].

### 3.3. Chimeric Compounds

In complex diseases, such as cancer, the simultaneous modulation of two or even multiple targets may be beneficial to the treatment of patients. Indeed, multitargeted drugs possess some advantages with respect to combinational therapies, such as predictable pharmacokinetic profile, absence of drug-drug interactions, and better patient compliance [49]. On the other hand, multitargeted therapy is often a very important cause of drug side effects due to interactions with anti-targets. In general, HDACIs are less potent against solid tumors with respect to their activity in haematological cancers. To obtain improved and wider anticancer efficacy, the design of chimeric HDACIs, including pharmacophoric elements directed against other targets has become a popular strategy.

**Figure 10:**
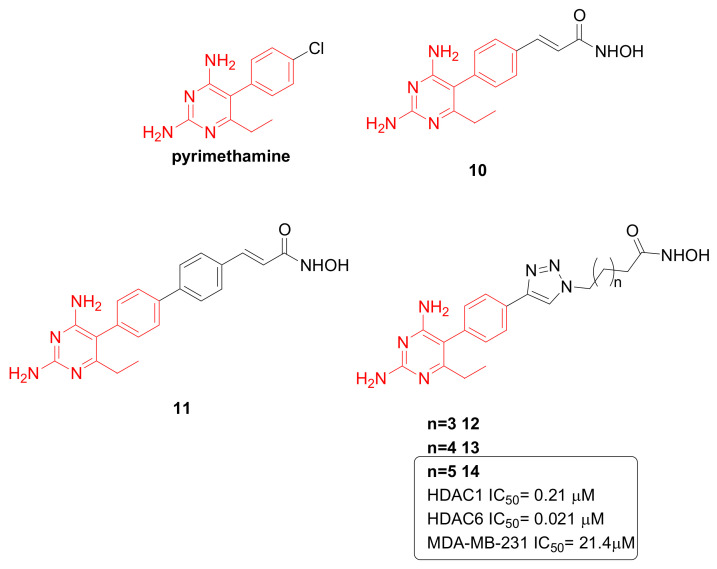
Chemical structures of HDAC/STAT3 inhibitors. Compound **14** had the ability to be directed quite selectively against HDAC6 and to impair TNBC progression.

STAT3 is an oncogenic transcription factor activated in many cancers, including TNBC [50], where it promotes cell proliferation and inhibits apoptosis by increasing the expression of target genes survivin, c-Myc, cyclin D1, B-cell lymphoma-2 (Bcl-2), and B-cell lymphoma-extra large (Bcl-xL). Starting from the observation that pyrimethamine is an inhibitor of STAT3, it was hypothesized that hydroxamate-based HDAC inhibitors bearing pyrimethamine as the CAP group (Figure 10) could be efficient anticancer agents [51]. Three different scaffolds were designed in which the pyrimethamine CAP group was spaced from the ZBG by means of a phenyl or biphenyl group (compounds **10**–**11**, Figure 10) or an aryl triazolyl group (compounds **12**–**14**, Figure 10). These molecules inhibited the HDAC isoforms 1, 6, and 8 with IC_50_ ranging from low nanomolar to micromolar, and compound **14** emerged as the most promising compound. In fact, it caused concentration-dependent upregulation of p-p38 and downregulation of Bcl-xL in MDA-MB-231 cells, confirming that an inhibition of STAT3 had occurred. Furthermore, this compound impaired TNBC cells proliferation with selectivity over non-tumoral VERO cells [51].

**Figure 11:**
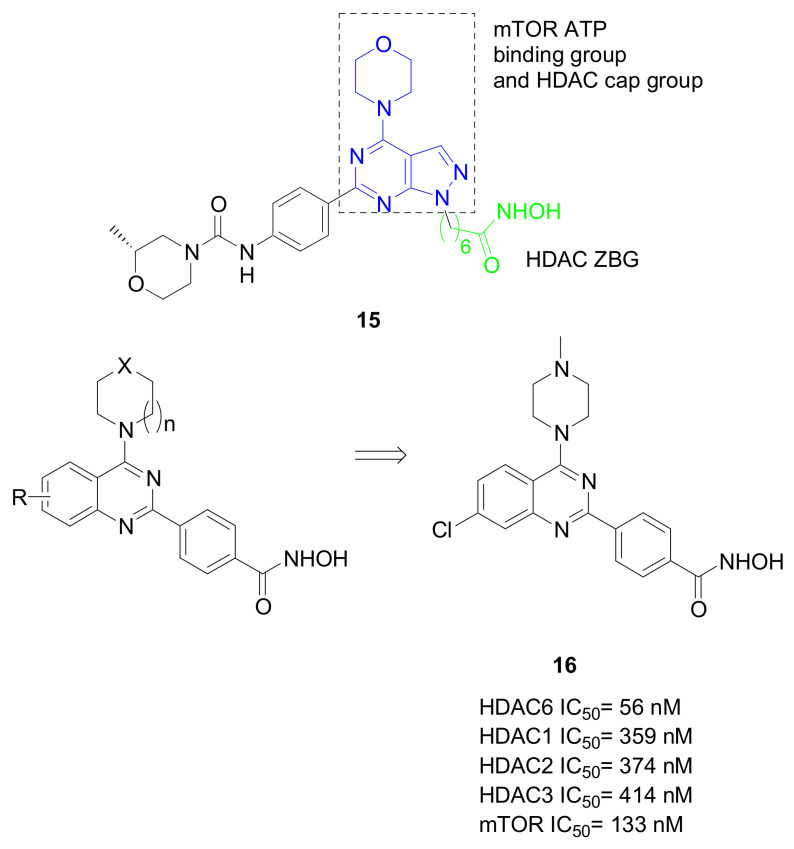
Chemical development of mTOR/HDAC inhibitors.

In analogy with this study, the synthesis and biological evaluation of dual mammalian target of rapamycin (mTOR)/HDAC6 inhibitors in TNBC cells was reported [52]. mTOR is a serine/threonine kinase playing essential roles in cell growth and survival, and its excessive signalling promotes the progression of different tumors [53]. Based on preliminary data on the antitumor effects of the first discovered dual mTOR/HDAC6 inhibitor against haematological malignancies (**15**, Figure 11) [54], aromatic hydroxamates containing a substituted pyrimidine as the pharmacophoric group directed against mTOR were prepared (Figure 11). The selected potent dual agent **16** had the ability to impair the proliferation of MDA-MB-231 cells, inducing autophagy and apoptosis and reducing cells migration as a consequence of the decreased phosphorylation and subsequent activation of mTOR.

**Figure 12:**
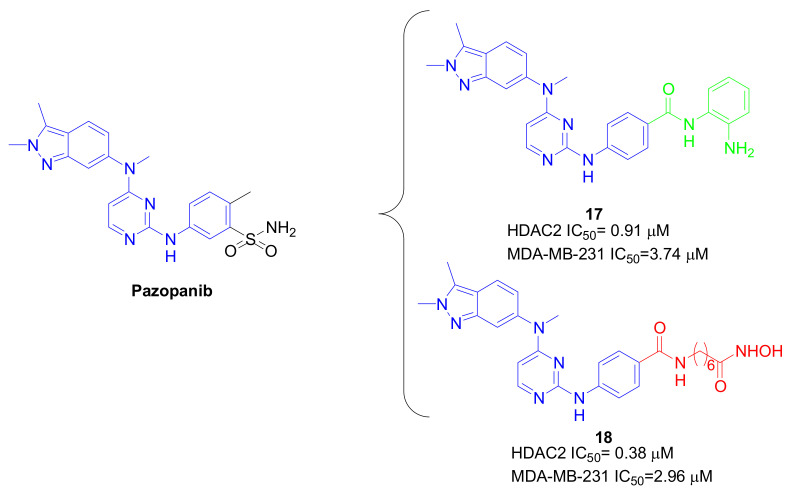
Chemical structures and biological activity of compounds **17** and **18**.

Furthermore, interesting results against TNBC cells proliferation were obtained from chimeric HDACIs bearing the Pazopanib core as the CAP group (Figure 8). Pazopanib is a multiple vascular endothelial growth factor (VEGF) inhibitor approved by the FDA for the treatment of advanced renal carcinoma and advanced soft tissue sarcoma in 2009 and 2012, respectively [55]. To overcome the drug resistance which occurs in treated patients, the combined therapy of Pazopanib with other drugs is recommended, with encouraging results when HDACIs are used as Vorinostat. On this basis, new Pazopanib-based benzamides and hydroxamates were designed (Figure 12) [56]. Compounds **17** and **18** emerged as the most promising ones, demonstrating an almost complete VEGF receptor-2 (VEGFR-2) inhibition rate at 0.2 μM, and potent HDAC2 inhibition (IC_50_ = 0.91 and 0.38 μM for **17** and **18**, respectively) together with antiproliferative effects against different solid tumor cell lines. In particular, these compounds compromised the viability of MDA-MB-231 cells with IC_50_ of 3.74 and 2.96 μM, respectively.

**Figure 13:**
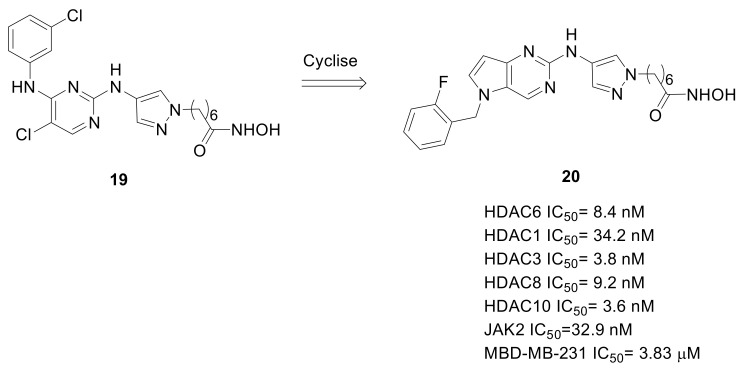
Chemical development of JAK/HDAC dual inhibitors: Structures of compounds **19** and **20**.

A further study confirming the usefulness of chimeric compounds to strengthen the therapeutic value of HDACIs against solid tumors, describes the development of dual Janus kinase (JAK)/HDAC inhibitors directed against TNBC [57]. Inhibition of JAKs is promising to impair cancer cells crosstalk and microenvironment, and the combination of JAK and HDAC pharmacophores is expected to overcome HDAC limitations against solid tumors. Therefore, starting from the leading scaffold of the reported JAK/HDAC dual inhibitor **19** (Figure 13), a novel series of pyrrolo[2,3-d]pyrimidine-based derivatives was synthesized. The adopted drug design allowed for the identification of different promising dual agents, and **20** (Figure 13) is the representative compound. Both in the MDA-MB-231 and BT-549 cell lines, **20** inhibits JAKs and Class I-IIb HDACs, simultaneously and quite selectively. This compound was a potent antiproliferative agent against different TNBC cell lines, and importantly it had the ability to inhibit tumor growth in an in vivo MDA-MB-231 mice xenograft model [57].

**Figure 14:**
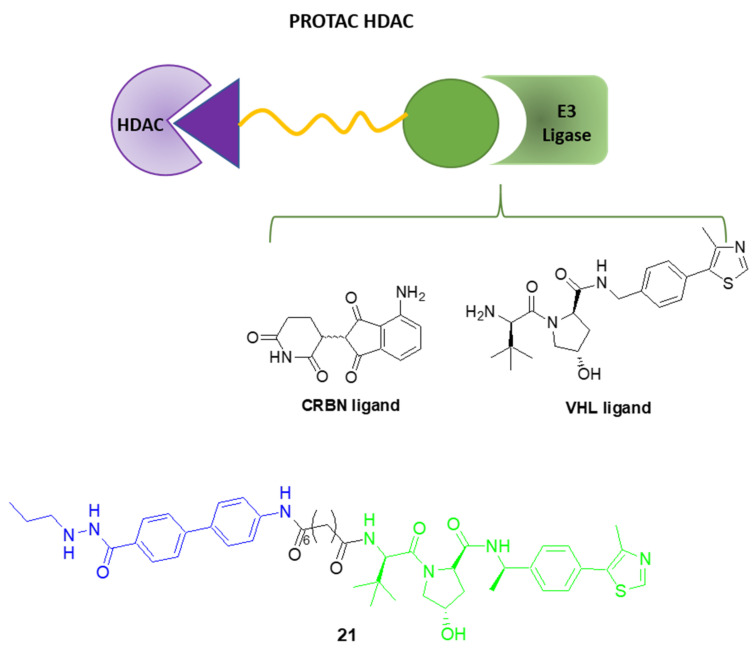
Schematic representation of PROTAC HDAC degraders and chemical structures of compound **21**.

Encouraging in vitro results against TNBC were also obtained by the so-called PROteolysis-TArgeting Chimeras (PROTACs) HDAC (Figure 14). They are bifunctional compounds showing a moiety that has the ability to bind to the E3 ubiquitin ligase, such as the cereblon (CRBN) and von Hippel–Lindau (VHL) ligands [58], and a HDAC binding group connected by a suitable linker. By forming a ternary complex E2 ligase:PROTAC:HDAC, they have the ability to trigger the ubiquitin-proteasome system (UPS), inducing the HDAC degradation [59,60]. Moreover, an interesting isoform selectivity can be achieved through the introduction of the appropriate HDAC binding moiety. For example, in compound **21**, (Figure 14) the Class-I selective HDAC inhibitor SR-3558 was linked by an alkyl chain to the VHL moiety and it was demonstrated that this compound dose-dependently induces HDAC3 degradation in the MDA-MB-468 cell line, suppressing the clonogenic growth of these cells, as well as of other breast cancer cell lines [61].

## 4. Conclusions

The development of HDACIs is an unquestionable success of the medicinal chemistry, which occurred despite the biological complexity of the target, and rapidly developed in the last 20 years. However, great efforts and progresses are required to validate HDAC as targets against TNBC. In the last 5 years, the drug discovery programs related to HDACIs pointed at the optimization of the well-accepted pharmacophoric model to obtain class and isoform selectivity. This approach reveals a promising potential toward the identification of effective HDACIs directed against TNBC progression, pointing to Class-I (HDAC1, 2, and 8, in particular) and Class-IIb (mainly HDAC6) selective agents. Moreover, major advances in the battle against TNBC could occur from chimeric agents, that, acting as dual mechanism compounds, represent a straightforward response to the necessity of combining HDACIs with different drugs to tackle TNBC and solid tumors in general. In this field, the PROTACs targeting HDACs are very recently emerging as a promising research area of new anticancer agents. Furthermore, some encouraging results were obtained from these molecules against TNBC, although only preliminary results are currently available, and more compounds and evidence are necessary to confirm their usefulness.

The present review has highlighted the most representative drug discovery studies addressing the outlined research front to obtain new anti-TNBC agents.

## Figures and Tables

**Table 1:** Classification and cellular function of HDACIs.

Class	HDAC Isoform	Cellular Function
I	1, 2, 3	nuclear lysine deacetylation
	8	fatty acid deacylation
IIa	4, 5, 7, 9	acetyl-lysine recognition
IIb	6	Cytoplasmatic lysine deacetylation
	10	polyamine deacylation
IV	11	fatty acid deacylation

**Table 2:** Summary of the main biological effects of HDACIs in TNBC cells.

HDACIs	Action	Ref.
Pan-HDACIs (Vorinostat, Panobinostat) and Class-I Selective (Entinostat)	Antiproliferative, pro-apoptotic, and immunomodulatory by: ↑p21 and p27 ↓FOXA1 ↓BCL2 ↑PD-L1 ↓DNA repair ↓MMP9	[7,21,23,24,28,29,30,31]
Class-I HDAC Selective (Entinostat)	ER− to ER+ conversion; compromise vasculogenic mimicry data	[8,25]
HDAC6 Selective (BAS-6)	Alteration of the glycolytic metabolism	[27]

## Data Availability

Not applicable.
